# Improvement the Healthcare Quality of Emergency Department after the Cloud-Based System of Medical Information-Exchange Implementation

**DOI:** 10.3390/healthcare9081032

**Published:** 2021-08-11

**Authors:** Ding-Chung Wu, Hong-Ling Lin, Chun-Gu Cheng, Chia-Peng Yu, Chun-An Cheng

**Affiliations:** 1 Department of Medical Records, Tri-Service General Hospital, National Defense Medical Center, Taipei 11490, Taiwan; wuperwu@gmail.com (D.-C.W.); lynnrainbow106@gmail.com (H.-L.L.); yu6641@gmail.com (C.-P.Y.); 2 School of Public Health, National Defense Medical Center, Taipei 11490, Taiwan; 3 Institute of Life Science, National Defense Medical Center, Taipei 11490, Taiwan; 4 Department of Emergency Medicine, Taoyuan Armed Force General Hospital, National Defense Medical Center, Taoyuan 32549, Taiwan; doc50015@yahoo.com.tw; 5 Department of Emergency Medicine, Tri-Service General Hospital, National Defense Medical Center, Taipei 11490, Taiwan; 6 Department of Emergency and Critical Medicine, Wan Fang Hospital, Taipei Medical University, Taipei 11696, Taiwan; 7 Department of Neurology, Tri-Service General Hospital, National Defense Medical Center, Taipei 11490, Taiwan

**Keywords:** medical information exchange, cloud-based system, healthcare quality

## Abstract

Background: The National Health Insurance has been implemented in Taiwan since 1995. The government established a medical information-exchange system to reduce duplicate medications and examinations, which have inhibited healthcare expenditures. The potential benefit of medical information exchange about healthcare quality in emergency departments (ED) was worthy of evaluating; Methods: The inquiry rate of cloud data for patients’ information in Taiwanese National Health Insurance Administration was defined as a factor, and the healthcare quality included the ratio of staying more than 48 h in the ED and the hospitalization rate within 8 h from ED by triage levels of 1, 2, and 3 in different levels of hospitals from 2013 to 2019. Poisson regression analysis was used to quantify time trends of the query rate of the MediCloud system, the rate of staying more than 48 h in ED, admission rate within 8 h in ED, and the effect of healthcare quality in ED after MediCloud system implementation; Results: The health information exchange decreased the rate of staying over 48 h in the ED of medical centers. It also improved the early hospitalization of urgent ED patients in regional hospitals; Conclusions: Through medical information exchange to understand patients’ current conditions, we can reduce crowding in the ED of medical centers and facilitate rapid hospitalization of urgent patients in regional hospitals. According to these findings, the government should establish medical information exchange to improve the healthcare quality of ED.

## 1. Introduction

The National Health Insurance (NHI) with a single insurer has been implemented in Taiwan since 1995, covering the majority of citizens and supporting complete medical care with higher accessibility and low payment, intended to address part of the burden and eliminate the economic barriers and protect medical rights with through lower payment [1]. The patients seek medical care in different medical institutions, and personal medical information is often scattered. It is difficult for doctors to fully recognize medical information of patients from a single-institution without exchange; it increases the risk of duplicate prescriptions or examinations, with increased health insurance expenditures.

All medical institutions adopt electronic declaration. The claim and payment systems are fully automated. The duplicate medications, because of drug interactions and toxicities, waste medical resources and endanger patients’ safety [2]. Repeat radiology examinations can cause radiation over-exposure, increasing cancer risk [3]. The NHIA promoted cloud technology to build a PharmaCloud system to improve the efficiency of medical care, the safety of medications, and healthcare in 2013 [4]. Each health care provider could query the patient’s recent records of medication and examinations within 3 months. There were some studies for attitudes of health providers, drugs and drug interaction, and duplicate medications with the aim of increasing safety through clinical decision-support technology that improves the safety of medications [2,5,6]. It was upgraded to the MediCloud system based on the needs of clinical practice, with reports of examinations and inpatient electronic health records added in 2016. The medical image exchange was established in 2018. The doctors must check the system to share images and avoid auditing before an arranged medical imaging. Therefore, patients do not request duplicate procedures; therefore, potential repeat image examinations are avoided, saving time and costs. [1,7].

The crowding of emergency departments (EDs) in medical centers are caused by increasing volumes of older patients, complexities, lack of inpatient resources, hospital staff shortage especially in nurses, non-urgent use, and holiday effect with outpatient as well as primary care. The convenience of healthcare insurance coverage and the habits of medical care search with lower payment in the EDs in Taiwan have caused overuse and crowding in EDs. It is worthy of evaluating the potential effect of policies on this issue.

Taiwan has entered into a super-aged society. Older patients have multiple illnesses and increased medical expenses. The lower severity patients with triage levels of 3 to 5 received emergency services of medical centers represent 85% in Taiwan [8]. The Ministry of Health and Welfare carried out the policies with reward by encouraging medical information exchange through upload and query, increasing the burden of milder disease treatment and strengthening the service capacity of primary medical institutions, thus promoted the hierarchical medical system. The association between medical information exchange and the healthcare quality of ED was, at the time, not fully known.

The purpose of this study is to access whether the inquiry of medical information by healthcare providers in the cloud system possibly improved the problems of the crowding of EDs with patients staying more than 48 h and early hospitalization from EDs within 8 h, according to different level hospitals and triage levels.

## 2. Materials and Methods

**Figure 1:**
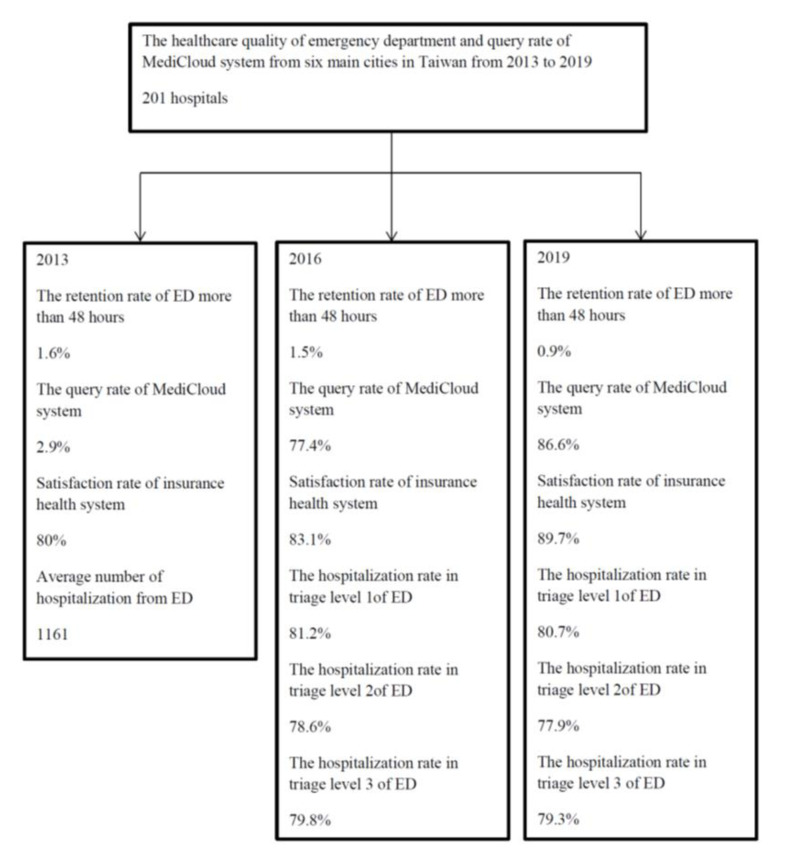
The flowchart of this study.

This research adopts a secondary data research method; the data source is the statistical data released by the NHIA, including the results of the public opinion on medical rights and interests from 2013 to 2019. The flow chart is shown in Figure 1.

The frequency of healthcare quality of EDs is recorded once a quarter from six main cities, including Taipei, New Taipei, Taoyuan, Taichung, Tainan, and Kaohsiung, which were screened. These areas represent a higher distribution of medical resources, higher access to medical treatment, and a higher degree of urbanization in Taiwan. The hospital levels are distinguished by medical centers, regional hospitals, and local hospitals.

This study does not require ethical approval, as it includes only information freely available in the public domain and the analysis of open-source datasets, where the data are properly anonymized.

The average annual inquiry rates of MediCloud data for patients’ medical information were collected year by year. The Ministry of Health and Welfare implemented the Triage Acuity Level of ED in 2010 to quickly verify the criticality and severity of ED patients’ conditions [9].The ratio of patients who stayed more than 2 days and the early hospitalization rate from ED by triage levels of 1, 2, and 3 were retrieved.

The outcome was defined as the healthcare quality information disclosure network retrieved from the historical data of the rate of staying more than 48 h in ED, and the hospitalization rate within 8 h from ED by triage level of 1, 2, and 3.

The data was filed in Excel 2010 version, and the Poisson regression analysis was used to quantify time trends of the query rate of the MediCloud system for the ratio of staying more than 48 h in ED, having an early admission rate within 8 h in ED, satisfaction rate of health insurance, and evaluated effect of healthcare quality of ED about emergency abuse and congestion in different levels of medical institutions after the policies of cloud medical information sharing. The impact of policy implementation on the quality of healthcare at all levels of medical institutions and triage levels. The statistical difference was defined as *p* < 0.05. The analysis used IBM SPSS version 22 (IBM SPSS Statistics for Windows, Version 22.0, Asia Analytics Taiwan Ltd., Taipei, Taiwan).

## 3. Results

**Figure 2:**
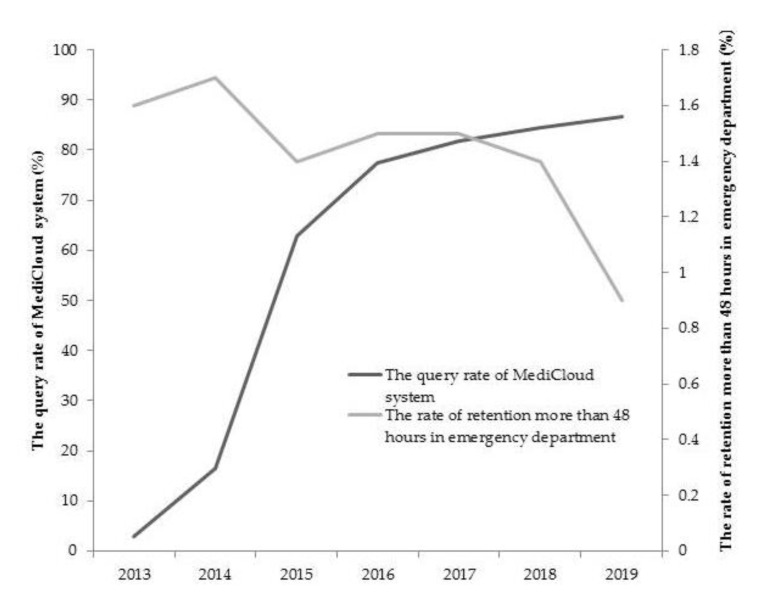
The time series of the annul query rate of MediCloud system and the retention rate of more than 48 h in emergency department.

There were 18 medical centers, 50 regional hospitals, and 133 local hospitals selected for healthcare quality in ED; this includes 40.61% (201/483) of all hospitals in Taiwan. The average value of each index and the trend changes over the years were observed. The emergency retention rate was greater than 48 h, with an average of 1.5%. It decreased significantly from 1.6% to 0.9% from 2013 to 2019 (*p* = 0.001) (Figure 2). On average, 81.0%, 78.2%, and 79.2% of admission rates were within 8 h in the first triage level, second triage level, and third triage level, respectively. There were a significant decrease of about 0.5% from 2016 to 2019 in triage level 1 (*p* = 0.001) and a significantly decrease of about 0.7% from 2016 to 2019 in triage level 2 (*p* < 0.001) (Table 1).

The average annual cloud inquiry rate increased from 2.9% in 2013, to 77.4% with the upgraded establishment of the MediCloud in 2016. The average annual query rate increased to 86.6% in 2019 (*p* < 0.001) (Figure 2). The average satisfaction rate of the patients about insurance health care was 82.7%, which increased from 80.0% in 2013 to 89.7% in 2019 (*p* < 0.001) (Table 1).

According to the public information provided by the NHIA, there were more hospitalizations at EDs in medical centers compared with regional hospitals and local hospitals (5044, 1935, 193, *p* < 0.001). The healthcare quality index observes the congestion of EDs in the past years. Overall, the number of cases transferred from EDs to wards has increased year by year. The average number of patients has increased from 1161 to 1341 patients (*p* = 0.0047) from 2013 to 2019 in all hospitals, 4782 to 5570 in medical centers (*p* < 0.001), and 162 to 253 in local hospitals (*p* < 0.001). The average number of patients in triage level 3 in local hospitals significantly increased (*p* < 0.001) (Table 2).

According to the hospital-level stratification analysis of healthcare quality in EDs, the rate of retention was greater than 48 h in the following amounts: 7.2% in medical centers, 1.6% in regional hospitals, and 0.4% in local hospitals (*p* < 0.001). We performed the stratified statistics at the hospital levels; the numbers of retention in ED for patients of medical centers was significantly decreased (*p* < 0.001) (Table 3).

The rate of admission within 8 h from ED for the first triage level were, on average, 59.6% in medical centers, 81.2% in regional hospitals, and 92.6% in local hospitals (*p* < 0.001). The admission rate for first triage level and second triage level within 8 h from ED were from 59.9% to 55.7% (*p* = 0.002), 57.0% to 53.8% (*p* < 0.001), and third triage from 52.5% to 51.4% (*p* = 0.01) in medical centers from 2016 to 2019. There was an increase of 76.6% to 78.8% in the early hospitalization rate of third-level triage EDs within 8 h in regional hospitals (*p* < 0.001) (Table 3).

The impact of the policy of cloud medical information sharing on the healthcare quality of ED at all triage and hospital levels was evaluated using Poisson regression model. Retention rate of more than 48 h was significantly improved in overall hospitals and medical centers, with 0.995 (95% confidence interval (C.I.): 0.991–1.00, *p* = 0.032) and 0.96 (95% C.I.: 0.964–0.975, *p* < 0.001). Admittance rate within 8 h was also significantly improved for triage level 3 in regional hospitals, with 1.276 (95% C.I.: 1.087–1.498, *p* = 0.003) (Table 4).

## 4. Discussion

In this study, we have demonstrated that the average annual inquiry rate of the cloud-based system for medical information exchange increased over 70% after the government promoted reward policies in 2016. The overall retention rate over 48 h significantly decreased in the EDs of medical centers. Patients’ stays were shortened as a result of appropriate hospitalizations and discharges after effective treatment in medical centers made possible by medical information exchange. The increased hospitalization within 8 h in triage 3 of regional hospitals was within available healthcare capacities; the medical information exchange brought about effective quality improvement.

Although it showed a lower inquiry rate initially [10], now, 87% of health providers will consult the system to avoid medications and examinations audit by NHI in 2019 after the implementation of the incentive policy with extra bonuses by government [7]. The daily check system for overlapped prescriptions was established for six types of chronic disease medications, including anti-hypertensive, anti-hyperlipidemia, diabetic, anti-schizophrenia, anti-depression, and sedation agents, in 2013; overlap was reduced by more than half from 2014 to 2018, with past studies finding a current 5.8% medication duplication rate, which saves about $US 13.44 million in expenditures for medicine [2,4].

The NHIA quickly integrated travel history and contact history to the MediCloud System, with information provided by the Immigration Department on 27 January 2020. When patients seek medical care through inputting the NHI insurance card, they are queried regarding travel and contact history. This system not only prompts health care providers to judge high-risk patients and adopt relevant infection-control measures but also provides a safe working environment. Though effective preventive strategies, it resulted in a lower infection rate and in-hospital infection in Taiwan. My Health Bank was constructed for sufficient primary care with health management and primary prevention in 2014, and it is able to check healthcare data stretching back three years. The numbers of uses were about 1.66 million in January 2020; its use is persistently increasing when patients access it to buy face masks for COVID-19 [11]. The assessment of patients’ medical records enhances their perceived control and has shown to reduce anxiety in diabetic studies [12]. The system allows the patient to be active participants in receiving their updated medical records, thereby promoting primary prevention through health management.

The medical image exchange has been provided since 2018. The expenditures of health insurance were reduced by about $US 3.48 million every month after instituted by decreasing repeat image studies [8]. The information and system quality directly affected satisfaction, and service quality was indirectly affected as trust was built in the cloud computing health care services as a mediator between healthcare providers [13]. The satisfaction of the NHI and MediCloud system from insurance citizens’ survey was 83.1% and 92% among insurance patients who agreed to spend about 1–2 min engaging in the systematic survey of doctors in 2016 [8].

There were 5004 intensive care unit (ICU) wards in six main cities. ICUs have the highest rate of use, with 83.88% in medical centers, 69.9% in regional hospitals, and 63.88% in local hospitals in 2018. The use rate of general wards was 83.29% in medical centers, 68.56% in regional hospitals, and 63.54% in local hospitals in 2018 [14]. The number of patients admitted to EDs increased in medical centers because the patients’ tendency to seek healthcare in the EDs of medical centers correlated with higher trust of medical care and examinations regarding severe health problems suffered by themselves and families. There were relatively serious medical conditions in medical centers. There was only below 60% of triage level 1–3 patients admitted to EDs within 8 h, representing a significant reduction for medical centers; it presented increasing delayed hospitalizations of critical patients in medical centers. The inpatient medical care in medical centers is used more often in Taiwan. The problem of crowding still exists in medical centers. In addition, the potential reason may be the critical patients are initially sent to the regional or local hospitals, but they will be referred upward to the medical centers after initial surveys and treatment due to inadequate facilities in lower hospital levels. The mean hospitalization of patients in triage level 3 in local hospitals increased from 211 to 302 (*p* < 0.001) between 2016 and 2019. In the past, studies noted the advantages of the NHI system, including high medical accessibility, low costs, and shorter waiting time. However, strict gatekeeper screening and improvement in healthcare quality will be important issues to be solved in future years [15].

This study showed that the adjustment of the burden policy of EDs with lower triage levels is still not enough to change the patients’ medical care-seeking behavior. Patients are often sent to EDs with acute diseases or new sick diseases; however, healthcare providers still need to perform the necessary detailed interview and examinations to assist in diagnosis and treatment. The inquiry of the MediCloud system can help healthcare providers to understand recent medical history and examinations within 3 months and access the patients’ disease references, then support differential diagnosis and treatment to improve patient care in EDs. The government promoted a two-way referral system to increase the patients’ willingness for downward referral. When the patients accept downward referral arrangements from medical centers to lower-level hospitals, the medical records could query in the MediCloud system from accepted hospitals to reduce the rejection of the healthcare provider.

As we have shown, the congestion of higher triage levels in ED of medical centers and the early admission rate to EDs of serious illness patients that need intensive care decreased in medical centers after the institution of medical-exchange inquiry. The usage rate of ICU in medical centers was around 80%, which is still not enough to meet the demand. Our study found there were available wards in lower-level hospitals. The early admission rate increased for urgency patients of regional hospitals after medical information exchange was performed long term, showing that the available medical capacities of regional hospitals could still accommodate a downward referral policy and increase patients’ urgent care. The mean hospitalization of patients increased in local hospitals after the sound specialist physician system was established and after governmental promotion. This was implemented to improve the care of critical patients as well as training in regional and local hospitals; it could more successfully perform the downward transfer to the ED of lower level hospitals by increasing the incentive to solve crowding in EDs of medical centers. The strategies for ED crowding improvement in medical centers included vertical integration strategic alliance with regional and local hospitals, patients’ diversion and downward referral system promotion, medical care staff shortage, increase in care facilities, and the availability of more critical wards in medical centers.

The research data are taken from the public information with the scope for healthcare quality monitoring of medical institutions by the NHIA. A total of 70% cost of medical care is spent in the main six cities [1]. When the emergency specialist was promoted in 1998, there were 1074 (69%) emergency specialists in six main cities of Taiwan, with 1556 overall during 2018 [14]. We used the data of ED from the main six cities to evaluate healthcare quality regarding the majority of EDs. The strength of this study lies in the data regarding the annual query rate of the MediCloud system; as the healthcare quality of the ED covers the majority of medical hospitals in Taiwan, it represented nearly fully the national population.

However, there were several limitations in this study. First, we surveyed the rate of retention of more than 48 h and hospitalization rate triage levels 1–3 within 8 h from NHIA, but there was a gap of admission situations, with information from the 8–48 h interval stage not available with inference. Second, as regards the crowding of ED in medical centers due to the patients’ freedom of medical care selection, we did not assess the attitudes of patients, for which further studies can develop an in-depth evaluation. Third, the relationship between MediCloud query and ED healthcare quality is by indirect evidence; it requires an association study to confirm in the future.

## 5. Conclusions

In conclusion, the cloud-based system of medical information exchange in Taiwan has been implemented several years, which have had a profound impact on the medical environment and system. The establishment of the cloud system with medical information-exchange potential improved the congestion of EDs in medical centers. There were some quality improvement for healthcare in medical centers and regional hospitals. The effect of healthcare quality for medical information exchange is worthy of being implemented in other countries. Our study could support evidence for other countries in order to adopt the policies of medical information exchange.

## Figures and Tables

**Table 1:** The percentage of each healthcare quality during 2013–2019 by Poisson regression.

Quality	Total	2013	2014	2015	2016	2017	2018	2019	*p*
Rate of retention more than 48 h in ED	1.5%	1.6%	1.7%	1.4%	1.5%	1.5%	1.4%	0.9%	0.001 *
The admittance rate within 8 h in ED in triage level 1	81.0%				81.2%	81.2%	80.8%	80.7%	0.001 *
The admittance rate within 8 h in ED in triage level 2	78.2%				78.6%	78.5%	77.5%	77.9%	<0.001 *
The admittance rate within 8 h in ED in triage level 3	79.2%				79.8%	79.0%	78.7%	79.3%	0.383
The quarry rate of MediCloud system	60.2%	2.9%	16.6%	62.8%	77.4%	81.9%	84.5%	86.6%	<0.001 *
Satisfaction rate of health insurance	82.7%	80.0%	79.8%	81.0%	83.1%	85.8%	86.5%	89.7%	<0.001 *

* *p* < 0.05.

**Table 2:** The average hospitalizations of patients in different hospital levels and triage levels.

	Total	2013	2014	2015	2016	2017	2018	2019	*p*
Cases admitted from ER									
Total	1239	1161	1205	1224	1251	1251	1317	1341	0.0047 *
Medical center	5044	4782	4954	4990	5162	5112	5138	5570	<0.001 *
Regional hospital	1935	1850	1908	1945	1979	1936	1981	1983	0.292
Local hospital	193	162	164	177	188	211	246	253	<0.001 *
Triage level 1									
Total	189				192	191	186	189	0.9905
Medical center	528				537	541	514	491	
Regional hospital	171				168	168	175	184	
Local hospital	29				25	29	32	40	
Triage level 2									
Total	506				511	491	514	513	0.8724
Medical center	1539				1547	1512	1578	1470	
Regional hospital	439				440	428	444	474	
Local hospital	71				64	67	79	85	
Triage level 3									
Total	1103				1082	1077	1139	1151	0.2688
Medical center	2924				2961	2902	2915	2904	
Regional hospital	1283				1260	1261	1314	1342	
Local hospital	248				211	237	284	302	

* *p* < 0.05.

**Table 3:** Rate of retention more than 48 h in emergency department from different hospital levels.

	Total	2013	2014	2015	2016	2017	2018	2019	*p*
The rate of retention more than 48 h									
Total	1.5%	1.6%	1.7%	1.4%	1.5%	1.5%	1.4%	0.9%	0.001 *
Medical center	7.2%	8.6%	8.5%	7.5%	6.5%	6.2%	6.3%	6.0%	<0.001 *
Regional hospital	1.6%	1.3%	1.7%	1.6%	1.8%	1.6%	1.5%	0.9%	0.237
Local hospital	0.4%	0.4%	0.4%	0.2%	0.5%	0.7%	0.4%	0.1%	0.672
The admittance rate within 8 h in triage level 1									
Total	81.0%				81.2%	81.2%	80.8%	80.7%	<0.001 *
Medical center	59.6%				59.9%	61.3%	58.4%	55.7%	0.002 *
Regional hospital	81.2%				81.3%	81.2%	81.1%	81.1%	<0.001 *
Local hospital	92.6%				93.3%	92.4%	91.9%	93.2%	0.754
The admittance rate within 8 h in triage level 2									
Total	78.2%				78.6%	78.5%	77.5%	77.9%	0.015 *
Medical center	55.5%				57.0%	56.5%	53.5%	53.8%	<0.001 *
Regional hospital	77.2%				77.6%	77.5%	76.6%	76.6%	<0.001 *
Local hospital	90.7%				91.0%	90.5%	90.5%	91.6%	0.321
The admittance rate within 8 h in triage 3									
Total	79.2%				79.8%	79.0%	78.7%	79.3%	0.383
Medical center	51.7%				52.5%	52.0%	50.8%	51.4%	0.01 *
Regional hospital	77.1%				76.6%	76.3%	77.9%	78.8%	<0.001 *
Local hospital	91.4%				93.1%	91.5%	90.0%	90.4%	<0.001 *

* *p* < 0.05.

**Table 4:** The effect of healthcare quality in emergency department after MediCloud system implementation.

	Relative Risk (95% Confidence Interval)	*p*
Retention rate more than 48 h (All)	0.995 (0.991–1.00)	0.032 *
Retention rate more than 48 h in MC	0.96 (0.964–0.975)	<0.001 *
Retention rate more than 48 h in RH	0.999 (0.993–1.006)	0.812
Retention rate more than 48 h in LH	1.000 (0.996–1.004)	0.983
Early admittance rate in Triage 1 (All)	0.943 (0.915–0.972)	<0.001 *
Early admittance rate in Triage 1 in MC	0.646 (0.429–0.971)	0.036 *
Early admittance rate in Triage 1 in RH	0.977 (0.972–0.982)	<0.001 *
Early admittance rate in Triage 1 in LH	0.95 (0.812–1.112)	0.521
Early admittance rate in Triage 2 (All)	0.903 (0.833–0.978)	0.012 *
Early admittance rate in Triage 2 in MC	0.667 (0.544–0.818)	<0.001 *
Early admittance rate in Triage 2 in RH	0.884 (0.831–0.940)	<0.001 *
Early admittance rate in Triage 2 in LH	1.039 (0.918–1.176)	0.545
Early admittance rate in Triage 3 (All)	0.949 (0.875–1.028)	0.199
Early admittance rate in Triage 3 in MC	0.857 (0.774–0.948)	0.003 *
Early admittance rate in Triage 3 in RH	1.276 (1.087–1.498)	0.003 *
Early admittance rate in Triage 3 in LH	0.719 (0.642–0.805)	<0.001*

* *p* < 0.05. MC, medical center; RH, regional hospital; LH, local hospital.

## Data Availability

The hierarchical medical care and effect of medical information exchange: https://www.nhi.gov.tw/Content_List.aspx?n=77E733B4D7F423AC&topn=787128DAD5F71B1A (accessed on 13 April 2020); The healthcare quality: https://www.nhi.gov.tw/AmountInfoWeb/TargetItem.aspx?rtype=2 (accessed on 13 April 2020).
